# Functional molecule guided evolution of MnO_*x*_ nanostructure patterns on N-graphene and their oxygen reduction activity†

**DOI:** 10.1039/c9ra04677a

**Published:** 2019-09-04

**Authors:** Nibedita Behera, Swarna P. Mantry, Biswaranjan D. Mohapatra, Rajesh K. Behera, Kumar S. K. Varadwaj

**Affiliations:** Department of Chemistry, Ravenshaw University Cuttack Odisha 753003 India skvardwaj@ravenshawuniversity.ac.in

## Abstract

In this work, we systematically followed the growth of MnO_*x*_ nanostructures on trimesic acid (TMA)/benzoic acid (BA) functionalised nitrogen doped graphene (NG) and studied their electrocatalytic activity towards oxygen reduction reaction (ORR). In these hybrid materials the MnO_*x*_ phase, their morphology and Mn surface valency were guided by the functional molecules, their concentration and the duration of reaction, which in turn significantly affected the ORR activity. During the growth in the presence of TMA, agglomerated nanostructures were formed at 2 h reaction, which transformed to well dispersed 4–7 nm particles at 6 h over a large area of NG. However, in the presence of BA, MnOOH nano-flecks were formed at 2 h and transformed to MnOOH nanowires and oval shaped Mn_3_O_4_ particles at 8 h of reaction. The valency of surface Mn on the different MnO_*x*_ nanostructures was ascertained by X-ray photoelectron spectroscopy (XPS). The ORR activity of samples were studied by cyclic voltammetry (CV) and rotating disc electrode (RDE) in alkaline medium. Among all the studied samples, the highest ORR activity with most efficient 4e^−^ transfer process is observed for TMA modified NG-MnO_*X*_ obtained at 6 h of reaction, which is due to its well dispersed nanostructure morphology.

## Abstract 2

In this work, we systematically followed the growth of MnO_*x*_ nanostructures on trimesic acid (TMA)/benzoic acid (BA) functionalised nitrogen doped graphene (NG) and studied their electrocatalytic activity towards oxygen reduction reaction (ORR).

## Graphical abstract

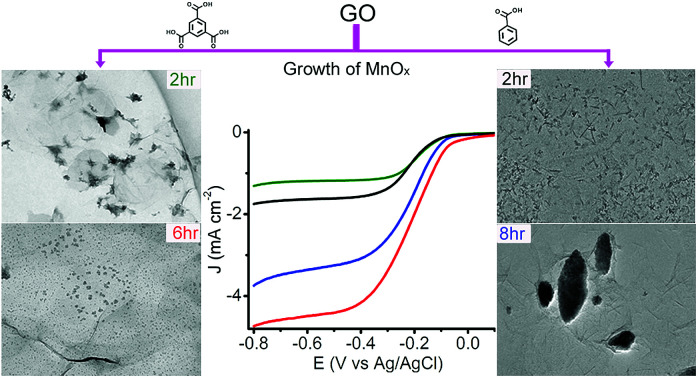

## Introduction

There is a large body of work from the last decade, focused on the growth of reduced graphene oxide (rGO) supported metal oxide nanostructures due to their applications in high performance electrocatalysts, sensors, battery materials, and supercapacitors.^1–4^ Metal oxide nanostructures anchored on rigid 2-dimentional rGO prevent agglomeration of nanoparticles, provide more exposed area at the electrolyte interface and show increased electrical conductivity, which is responsible for their enhanced electrocatalytic activity.^5–8^ These hybrid materials are synthesized in a one or two step process in which few layered GO is reduced and loaded with metal oxide nanostructures.^9–12^ Metal oxides preferably nucleate and anchor at N or O sites on doped partially reduced graphene.^13–15^ Moreover, studies on the growth of metal oxides on carbon nanotube (CNT) demonstrate that particles prefer to nucleate at junctions of CNT or O containing functional groups.^16,17^ Therefore, this site specific nucleation ensures the growth of small nanoparticles on graphene. In recent years, there has been growing interest in the study of transition metal oxide @ graphene/CNT hybrid structures for electrocatalytic applications.^18–23^ Such hybrid materials show enhanced ORR activity as compared to their individual counterparts, which in turn is comparable to that of commercially available Pt/C.

In previous studies, hybrid materials are synthesized consisting nanostructures of MnO_*x*_/MnO/Mn_3_O_4_/MnO_2_ and NG/CNT.^9,19–23^ However, the optimum electrocatalytic activity of these hybrid structures can be realized by development of methods to control phase, shape and distribution of oxide nanostructures on NG. In solution phase synthesis, it has been observed that there is random distribution of nanostructures and only in few reports specific shaped nanoparticles could be achieved.^15,24–26^ Qiao *et al.* reported selective growth of sphere, cube and ellipsoid shaped Mn_3_O_4_ nanostructures on mesoporous NG and the ellipsoid particles showed highest ORR activity.^26^ Dai *et al.* demonstrated selective growth of plate and rod shaped oxide/hydroxide nanostructures of Fe, Co & Ni on graphene with different degrees of oxidation.^15^ However, in solution phase synthesis no effort has been made to understand the gradual evolution of different structural patterns on NG in a reaction medium. On the other hand, NG@Mn_3_O_4_ hybrid materials prepared by different methods show varying ORR activity.^22,23,26,27^ The electrochemically and solution phase grown NG@Mn_3_O_4_ by Bikkarolla *et al.* and Duan *et al.* respectively show better activity in comparison to other such samples.^9,22,23^ Although, enhanced ORR activity for these hybrid materials is attributed to C–N–M or C–O–M covalent linkage at the metal oxide–graphene interface such variations in activity may be addressed on systematically studying the evolution of MnO_*x*_ nanostructures on graphene.^1,23,27^

Indeed, MnO_*x*_ exists in wide verity of crystal structures and they show different catalytic activity towards ORR; while α-MnO_2_ shows the highest activity among different polymorphs of MnO_2_, the activity of other oxides follow the trend, Mn_5_O_8_ < Mn_3_O_4_ < Mn_2_O_3_ < MnOOH.^20,21,28–30^ It has also been observed that the surface Mn valency in MnO_*x*_ samples affects their ORR activity.^31–33^ In general, the presence of more amount of higher valence Mn *i.e.* Mn(iv) and Mn(iii) on the oxide surface augment the ORR activity.^31^ The presence of Mn(iv)/Mn(iii) couple in MnO_2_ and MnOOH, which act as a mediator for charge transfer to molecular oxygen, is believed to facilitate ORR activity.^32,33^

Furthermore, till date no effort has been made to control the distribution of oxide nanostructures on NG in solution phase. In solution phase growth of hybrid structures, uneven concentration of oxide nanostructures are observed on NG, which may be due to nanoparticle nucleation at the randomly distributed defect sites on NG.^5,9,26^ The covalent or non-covalent molecular functionalization of NG surface is one of the approaches to overcome this difficulty.^34,35^ The covalent functionalization on graphene alters the structure and electronic properties of graphene.^36^ In contrast, non-covalent functionalization tunes the electronic property and charge distribution on graphene without affecting its planner structure.^37–42^ In a previous study, we showed that on non-covalent functionalization of NG with molecules having electron withdrawing groups such as pyrene butyric acid or benzoic acid significantly enhance the ORR activity.^43^ However, no effort has been made to grow any metal oxide nanostructure on functionalized graphene, which is expected to tune the growth process and may give better control over its shape, size, phase and distribution. This in turn would help in designing such heterostructures with enhanced ORR activity.

Herein, we study the evolution of MnO_*x*_ nanostructures on TMA or BA functionalized NG in which the graphene oxide reduction, N-doping, molecular functionalization and MnO_*x*_ deposition are achieved in a single pot reaction. It has been observed that nanoparticle size, shape and distribution on the graphene sheet along with phase of MnO_*x*_ formed are determined by the functional molecules, their concentration and duration of reaction. In the early stage of reaction, agglomerated nanostructures or flecks like structures of MnO_*x*_ are observed on NG. Highly uniform distribution of 4 to 7 nm MnO_*x*_ particles on NG is only achieved through TMA modification after 6 h of reaction. Although MnOOH is the only phase formed in TMA, irrespective of its concentration and reaction time, formation of additional Mn_3_O_4_ phase with larger particle size is observed in BA at longer reaction time. We studied the electrocatalytic activity of the hybrid samples towards ORR and observed significant variation in activity for samples grown in different reaction conditions. The sample with well distributed MnO_*x*_ nanostructures on TMA modified NG showed highest ORR activity in terms of onset potential, limiting current density and electron transfer number.

## Experimental

### Synthesis of TMA@NGMnO_*x*_ and BA@NGMnO_*x*_ hybrid catalysts

First, Graphene oxide dispersion was prepared from graphite flakes (Sigma-Aldrich) by a modified Hummer's method.^43^ In a typical synthesis, 10 ml of graphene oxide (GO) dispersion in ethanol (2 mg ml^−1^), 50 mg TMA (Alfa Aesar)/BA (Himedia) and 5 ml of 30% NH_4_OH (Himedia) were mixed with 20 ml of 1,4-butanediol (Spectrochem) to produce a homogeneous solution. Then under reflux condition, the temperature of the solution was set at 180 °C. At this elevated temperature, 2 ml of 0.2 mM KMnO_4_ (Himedia) solution was dropwise injected into the reaction mixture for about 10 minutes and the reaction was allowed to continue. Part of the reaction mixture was withdrawn at different time intervals (2, 6, 8 and 12 h) and each one was separately centrifuged and washed with copious amount of distilled water and ethanol. The TMA functionalized samples collected after 2, 6, 8 & 12 h were denoted as TMA@NGMnO_*x*_/2, TMA@NGMnO_*x*_/6, TMA@NGMnO_*x*_/8 and TMA@NGMnO_*x*_/12 respectively, whereas BA functionalized samples were denoted as BA@NGMnO_*x*_/2, BA@NGMnO_*x*_/6, BA@NGMnO_*x*_/8 and BA@NGMnO_*x*_/12 respectively.

In another experiment, same reaction was carried out with 25 mg of TMA and the samples collected after 4, 8 & 12 h of reaction were named as 0.5TMA@NGMnO_*x*_/4, 0.5TMA@NGMnO_*x*_/8 and 0.5 TMA@NGMnO_*x*_/12 respectively.

### Physical characterization

The morphologies of the prepared samples were characterized by transmission electron microscopy (TEM, JEOL JEM 2010) with an operating voltage 200 kV. The crystal structure of the samples was determined by Ultima IV RIGAKU advance X-ray diffractometer with Cu Kα radiation at a scan rate of 2° min^−1^. The measurements were done in the 2*θ* range of 10° to 70°. The elemental analysis and oxidation state of Mn in the samples were done with X-ray photoelectron spectroscopy (XPS), PHI 5000 Versa ProbII, FEI Inc. Spectrometer. Here radiation Al Kα was used as X-ray source for the excitation. Binding energies were corrected using the C 1s peak at 284.8 eV as standard. Raman spectra was employed to characterize the structural information of the materials and recorded with Horiba Lab RAM HR confocal micro Raman system with excitation source 532 nm diode pumped solid state.

### Electrochemical measurements

All the electrochemical studies were performed with AUTOLAB potentiostat/galvanostat (PGSTAT204, Netherlands) workstation at an ambient temperature. The electrochemical cell consisted of three electrodes (i) Ag/AgCl as reference electrode (ii) Pt wire as counter electrode (iii) sample loaded glassy carbon (GC) electrode as working electrode. The GC working electrode was polished with 0.05 μm alumina powder, washed with lots of distilled water and dried in a desiccator. The catalyst ink was prepared by mixing 5 mg of sample with 2 ml isopropanol, 3 ml distilled water and 25 μl Nafion (Sigma-Aldrich) solution. The ink was sonicated for 15 min to prepare a homogeneous mixture and 12 μl ink solution was drop casted on the surface of the working electrode (GC, diameter 3 mm) and dried in the desiccator. CV measurements were done in 0.1 M KOH electrolyte with a scan rate of 10 mV s^−1^. The electrolyte was purged with pure N_2_ and O_2_ gas prior to every measurement for 30 minutes. For RDE the O_2_ atmosphere was maintained throughout the test. The voltammograms were recorded in the potential range of 0.2 to −0.8 V *vs.* Ag/AgCl.

## Results and discussion

### Effect of TMA functionalization

**Fig. 1:**
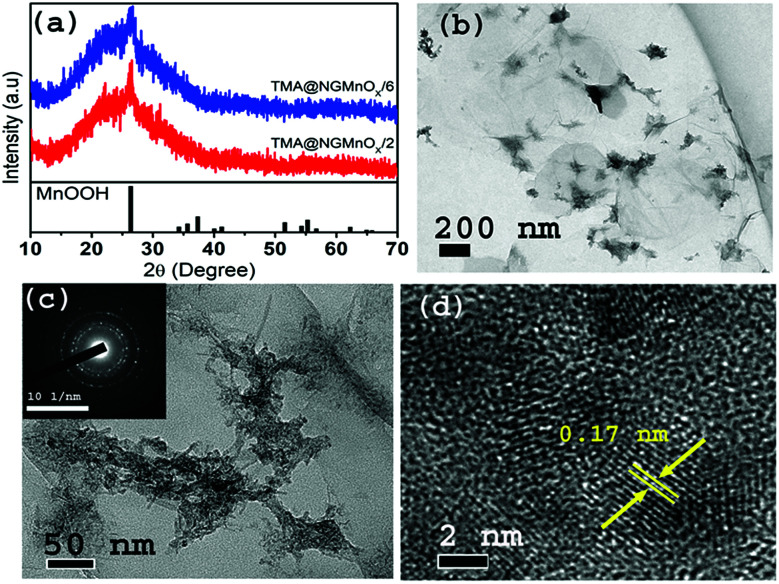
(a) XRD patterns of TMA@NGMnO_*x*_/2 and TMA@NGMnO_*x*_/6. TEM images of TMA@NGMnO_*x*_/2 with (b) low magnification and (c) high magnification and inset shows SAED pattern of TMA@NGMnO_*x*_/2. (d) The HRTEM image of TMA@NGMnO_*x*_/2 showing lattice fringes.

The XRD patterns of TMA@NGMnO_*x*_/2 and TMA@NGMnO_*x*_/6 in Fig. 1a show a broad hump centering around 22.5° (*d* = 3.94 Å) and a single peak at 26.4° (*d* = 3.36 Å). The broad hump may be attributed to the interlayer spacing of rGO and the peak may be assigned to highest intensity (110) diffraction peak of orthorhombic MnOOH (JCPDS PDF 74-1842). The absence of any other peak from MnOOH and broad nature of the single peak indicate poor crystallinity of the nanostructures. TEM images of TMA@NGMnO_*x*_/2 at two different magnifications in Fig. 1b and c show agglomerated MnO_*x*_ network structures without any definite shape. The HRTEM image in Fig. 1d shows spherical nanoparticles of ∼5 nm diameter and each particle has lattice fringes with spacing ∼0.17 nm corresponding to (220) plane from MnOOH. The SAED pattern (inset Fig. 1c) shows overlapping dot and ring patterns suggesting agglomerated single crystalline and polycrystalline nanostructures on TMA-NG.

**Fig. 2:**
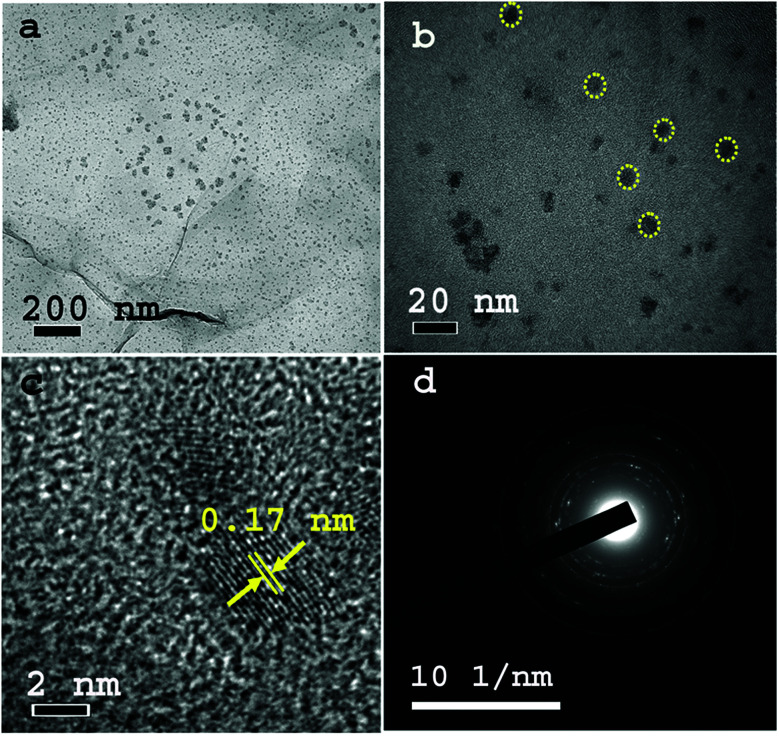
TEM images of TMA@NGMnO_*x*_/6 with (a) low magnification and (b) high magnification. (c) The HRTEM image of TMA@NGMnO_*x*_/6 showing lattice fringes and (d) SAED Pattern of TMA@NGMnO_*x*_/6.

In contrast, the TEM images of TMA@NGMnO_*x*_/6 in Fig. 2a and b at two different magnifications show uniformly distributed spherical MnO_*x*_ nanoparticles over a large area of graphene surface. The histogram for particle size distribution in. Fig. S1† shows that the particle size varies in the range of 3 to10 nm with maxima at 4–5 nm. The HRTEM and SAED analysis of TMA@NGMnO_*x*_/6 in Fig. 2c and d show similar single crystalline structure as that of TMA@NGMnO_*x*_/2. It may be noted that although the phase and crystallinity as observed from XRD analysis (Fig. 1a) almost remain unchanged, the particle distribution and shape has gone through a significant change with increase in reaction time. However, on further increasing the reaction time to 8 h, the TEM micrograph shows reduced density of nanostructures on NG & in 12 h sample only bare NG surface is observed (Fig. S2†). The sharp peak in XRD spectra due to MnOOH is also absent in 12 h sample. Therefore, we ascertain that initially agglomerated particles precipitate on TMA functionalised NG, subsequently excess TMA in the solution facilitate dissolution of the nanostructures. This process of dissolution and re-precipitation helps in formation of uniformly distributed nanostructures on NG. However, beyond 6 h in refluxing condition, solvent lose may have led to increase in TMA concentration in the reaction medium, which results in faster dissolution of nanostructures. Therefore, drastically reduced concentration of MnO_*x*_ nanostructures is observed in TMA@NGMnO_*x*_/8 and TMA@NGMnO_*x*_/12.

### Effect of TMA concentration

**Fig. 3:**
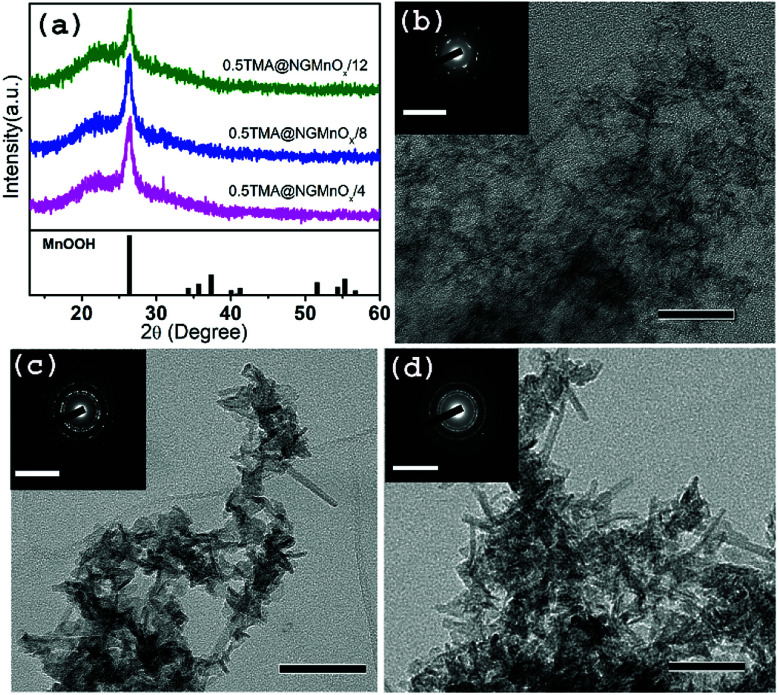
(a) The XRD patterns of 0.5TMA@NGMnO_*x*_/4, 0.5TMA@NGMnO_*x*_/8 and 0.5TMA@NGMnO_*x*_/12. TEM images of (b) 0.5TMA@NGMnO_*x*_/4 (c) 0.5TMA@NGMnO_*x*_/8 and (d) 0.5TMA@NGMnO_*x*_/12 and the insets show corresponding SAED patterns (scale bar for main figure: 50 nm and for inset 10 nm^−1^).

To study the growth of MnO_*x*_ in a decreasing rate of MnO_*x*_ dissolution, a reaction is carried out with TMA concentration reduced to half of the original amount. The XRD and TEM studies for heterostructures isolated at different time intervals (4, 8 &12 h) are shown in Fig. 3. The XRD patterns (Fig. 3a) show a single peak at 26.42° (*d* = 3.36 Å) similar to that observed in Fig. 1a and may be attributed to (110) diffraction peak of orthorhombic MnOOH (JCPDS PDF 74-1842). However, it is significant to note that, TEM images of 8 and 12 h samples show MnO_*x*_ nanostructures all over NG (Fig. S3†) and there is no remarkable dissolution with portions of bare NG as observed in TMA@NGMnO_*x*_/8 and TMA@NGMnO_*x*_/12 (Fig. S2†). Moreover, on reduction of TMA concentration none of the samples show well distributed nanostructures as observed in TMA@NGMnO_*x*_/6. The TEM micrographs of 8 and 12 h samples (Fig. 3c and d) show almost similar elongated nanostructures, which indicates that with decreased TMA concentration MnO_*x*_ dissolution is no more the dominant factor in controlling morphology of MnO_*x*_ on NG. It corroborates the fact that TMA not only functionalize the surface of graphene but also above a certain concentration in reaction medium helps MnO_*x*_ dissolution, which is necessary for getting uniform distribution of nanoparticles.

### Effect of BA functionalization

**Fig. 4:**
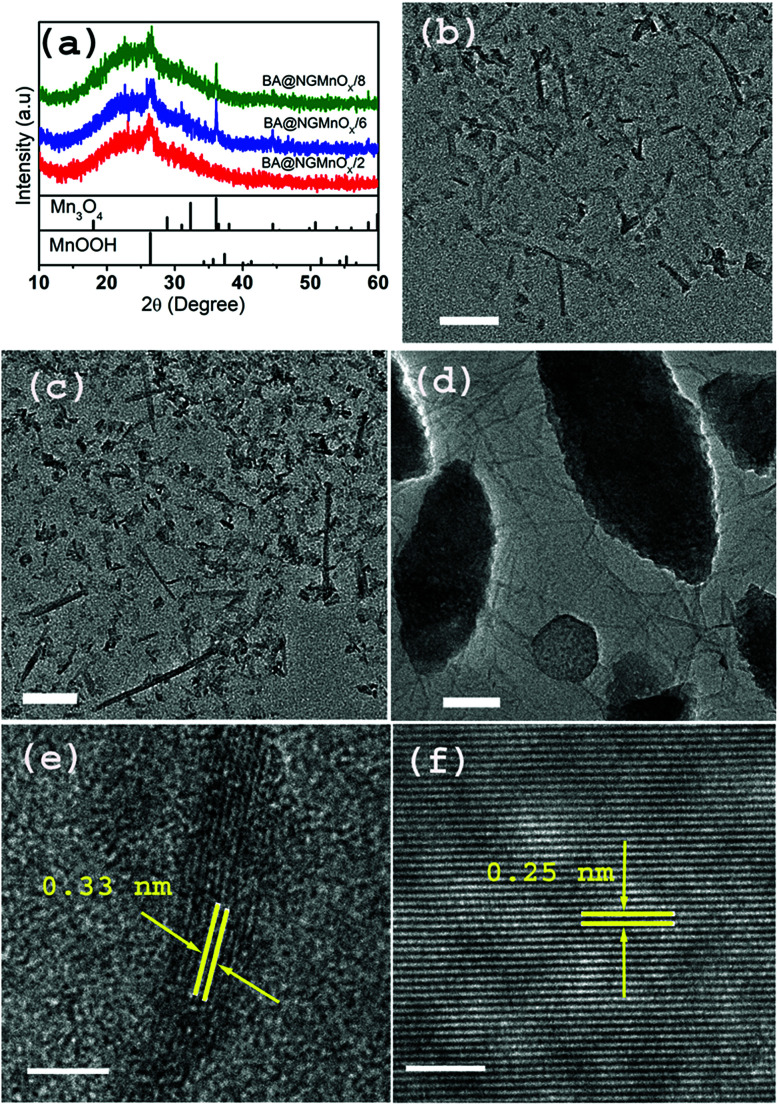
(a) The XRD patterns of BA@NGMnO_*x*_/2, BA@NGMnO_*x*_/6 and BA@NGMnO_*x*_/8. TEM image of (b) BA@NGMnO_*x*_/2, (c) BA@NGMnO_*x*_/6 and (d) BA@NGMnO_*x*_/8 (scale bar: 50 nm). HRTEM images of (e) BA@NGMnO_*x*_/8 wire and (f) BA@NGMnO_*x*_/8 oval structures showing lattice fringes (scale bar: 5 nm).

In order to investigate the role of different functional molecules on the morphology of the MnO_*x*_ nanostructures a reaction is performed taking BA as the functional molecule. Fig. 4 shows XRD patterns and TEM micrographs of BA@NGMnO_*x*_/2, BA@NGMnO_*x*_/6 and BA@NGMnO_*x*_/8. The XRD patterns of all the three samples in Fig. 4a show a broad hump centering around 22.5° (*d* = 3.94 Å) due to NG and a peak at 26.4° (*d* = 3.36 Å) which may be assigned to MnOOH (JCPDS PDF 74-1842). However, the patterns for BA@NGMnO_*x*_/6 & BA@NGMnO_*x*_/8 show additional peaks at 30.9°, 39.1° and 44.4° (*d* = 2.88, 2.48 and 2.03 Å respectively) which may be assigned to tetragonal Mn_3_O_4_ (JCPDS PDF 24-0734). Here it may be noted that during growth in presence of BA/TMA the same MnOOH is formed after 2 h of reaction time; however, on increase in the reaction time to 6 and 8 h Mn_3_O_4_ is formed along with MnOOH in presence of BA. It seems the reducing atmosphere in the polyol medium did not allow the formation MnO_2_ phase in the present method. But separation of GO reduction and MnO_*x*_ growth may allow us to get MnO_2_ nanostructures on NG.

The TEM images for BA@NGMnO_*x*_/2 and BA@NGMnO_*x*_/6 (Fig. 4b and c) show flecks and rod-shaped nanostructures, but a close observation revealed that the nanorods in BA@NGMnO_*x*_/6 are of remarkably longer length and width.

However, the TEM image for BA@NGMnO_*x*_/8 (Fig. 4d) shows completely different microstructures, which consist of almost ∼5 nm diameter nanowires and oval shaped bigger particles. The HRTEM images of nanowires and oval structures (Fig. 4e and f) show distinctly different lattice fringes with inter planer distance at 0.33 and 0.25 nm respectively, which may be attributed to (110) planes of MnOOH and (211) planes of Mn_3_O_4_ respectively. However, no further change in the microstructure could be observed on increasing the reaction time to 12 h (Fig. S4†).

**Fig. 5:**
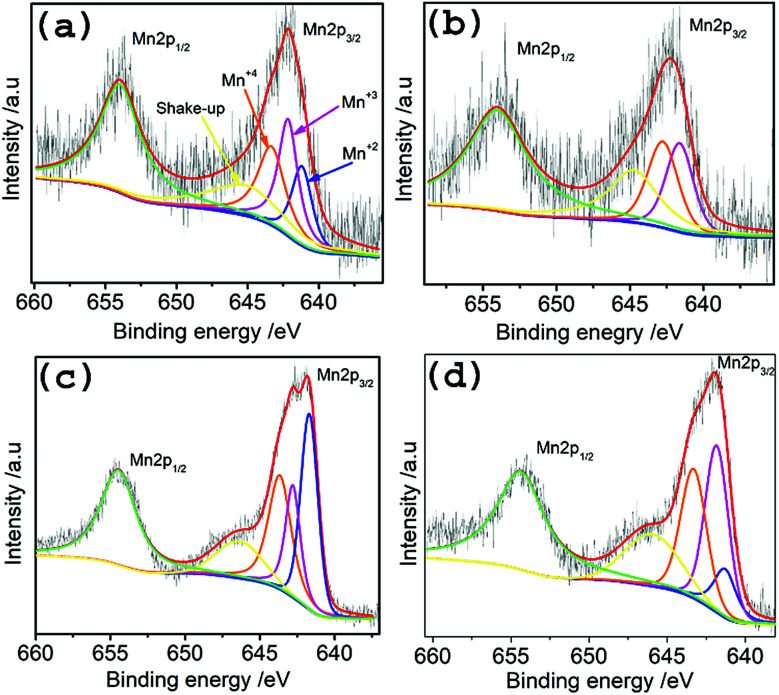
High resolution deconvoluted XPS spectra for Mn-2p region of (a) TMA@NGMnO_*x*_/2, (b) TMA@NGMnO_*x*_/6, (c) BA@NGMnO_*x*_/2 and (d) BA@NGMnO_*x*_/8.

To investigate the surface chemistry and Mn oxidation states in the hybrid materials, XPS measurements were done on TMA@NGMnO_*x*_/2, TMA@NGMnO_*x*_/6, BA@NGMnO_*x*_/2 and BA@NGMnO_*x*_/8. The XPS survey spectrum for TMA@NGMnO_*x*_/6 shows the presence of manganese, carbon, oxygen and nitrogen (Fig. S5†) in the sample. The amount of nitrogen are 2.4, 3.4, 5.6, 2.2 atomic percentage in TMA@NGMnO_*x*_/2, TMA@NGMnO_*x*_/6, BA@NGMnO_*x*_/2 and BA@NGMnO_*x*_/8 respectively. The N 1s peak in the survey spectra for NG, BA@NG, BA@NGMnO_*x*_/8 and TMA@NGMnO_*x*_/6 are shown in Fig. S6.† The shift in binding energy for the N peak of BA@NGMnO_*x*_/8 & TMA@NGMnO_*x*_/6 as compared to that of NG and BA@NG shows that MnO_*x*_ nanostructures could have grown on N sites. The Mn 2p core level scans in Fig. 5 for each of the hybrid material show two peaks corresponding to the binding energies of Mn 2p_3/2_ and peaks corresponding to the binding energies of Mn 2p_3/2_ and Mn 2p_1/2_. These spin–orbit doublets are present with a binding energy gap of 11.5 ± 0.1 eV. The deconvolution of Mn 2p_3/2_ spectra for all samples display four peaks with binding energies of 640.86, 641.92, 643.09 and 644.92 eV, which corresponds to Mn(ii), Mn(iii), Mn(iv) and the shakeup peak, respectively. This shakeup peak, noticeable on the higher binding energy (lower kinetic energy) side of the Mn 2p in the XPS spectra, originates from the charge transfer from outer electron shell to an unoccupied orbit with higher energy during the photoelectron process.^31^Table 1 contains the relative atomic percentage of different Mn oxidation states (Mn(ii), Mn(iii), Mn(iv)) in each hybrid sample as derived from their respective XPS spectrum. It shows that the amount of Mn(iv) and Mn(iii) is highest in TMA@NGMnO_*x*_/6 among all the studied samples. The Raman spectra of TMA@NGMnO_*x*_/2, TMA@NGMnO_*x*_/6, BA@NGMnO_*x*_/2, BA@NGMnO_*x*_/6 and BA@NGMnO_*x*_/8 show characteristic D and G bands for graphene at 1350 and 1592 cm^−1^ respectively (Fig. S7†). The *I*_D_/*I*_G_ ratio which is a measure of the defect concentration on graphene substrate, is ∼1 for all the samples.

**Table 1:** Relative atomic percentage of Mn(ii), Mn(iii), and Mn(iv) in various hybrid catalysts

Catalysts	Mn(ii)	Mn(iii)	Mn(iv)
TMA@NGMnO_*x*_/2	24.53%	38.44%	37.03%
TMA@NGMnO_*x*_/6	4.12%	46.3%	49.58%
BA@NGMnO_*x*_/2	39.9%	23.78%	36.32%
BA@NGMnO_*x*_/8	15.25%	45.22%	39.53%

### Electrochemical studies

**Fig. 6:**
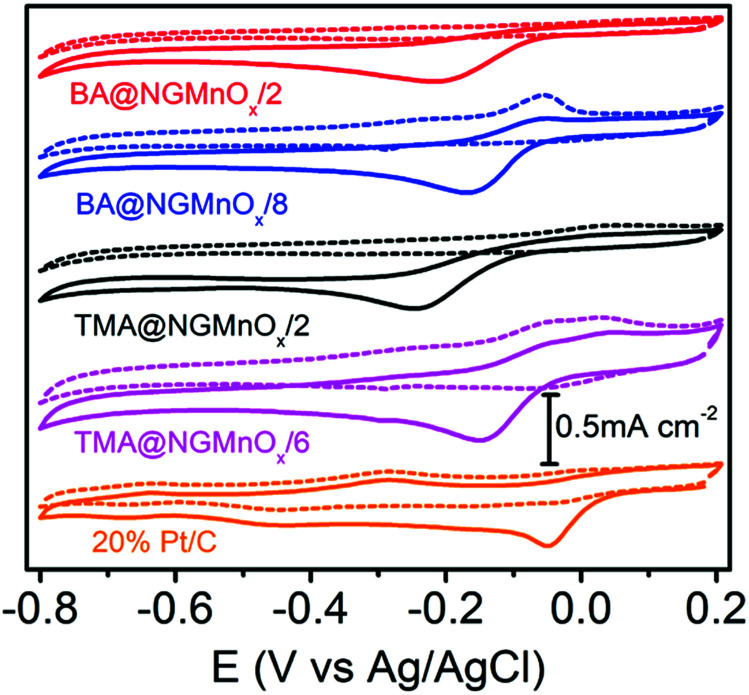
CV curves of TMA@NGMnO_*x*_/2, TMA@NGMnO_*x*_/6, BA@NGMnO_*x*_/2, BA@NGMnO_*x*_/8 and Pt/C in N_2_ (dotted curves) and O_2_ (solid curves) saturated 0.1 M KOH solution at a scan rate of 10 mV s^−1^.

Fig. 6 shows the CV curves for TMA@NGMnO_*x*_/2, TMA@NGMnO_*x*_/6, BA@NGMnO_*x*_/2 and BA@NGMnO_*x*_/8 in N_2_ and O_2_ saturated 0.1 M KOH electrolyte. The CV curves in N_2_ saturated electrolyte (dotted lines) for TMA@NGMnO_*x*_/2 and BA@NGMnO_*x*_/2 are featureless, but that for TMA@NGMnO_*x*_/6 and BA@NGMnO_*x*_/8 show peaks due to Mn(ii)/Mn(iii) or Mn(iii)/Mn(iv) redox transitions. However, in O_2_ saturated electrolyte (solid curves), an additional reduction peak appears due to oxygen reduction, for each of the catalyst sample during the cathodic sweep. The ORR onset and peak potential for TMA@NGMnO_*x*_/2 (−0.07 V, −0.24 V), TMA@NGMnO_*x*_/6 (−0.04 V, −0.14 V), BA@NGMnO_*x*_/2 (−0.072 V, −0.21 V) and BA@NGMnO_*x*_/8 (−0.055 V, −0.24 V) from the CV curves show that the ORR activity depends on the functional molecule as well as reaction time. The onset potential for the catalysts follows the order TMA@NGMnO_*x*_/6 > BA@NGMnO_*x*_/8 > TMA@NGMnO_*x*_/2 > BA@NGMnO_*x*_/2. The CV curve for the state of art 20wt% Pt/C catalyst is shown for comparison.

**Fig. 7:**
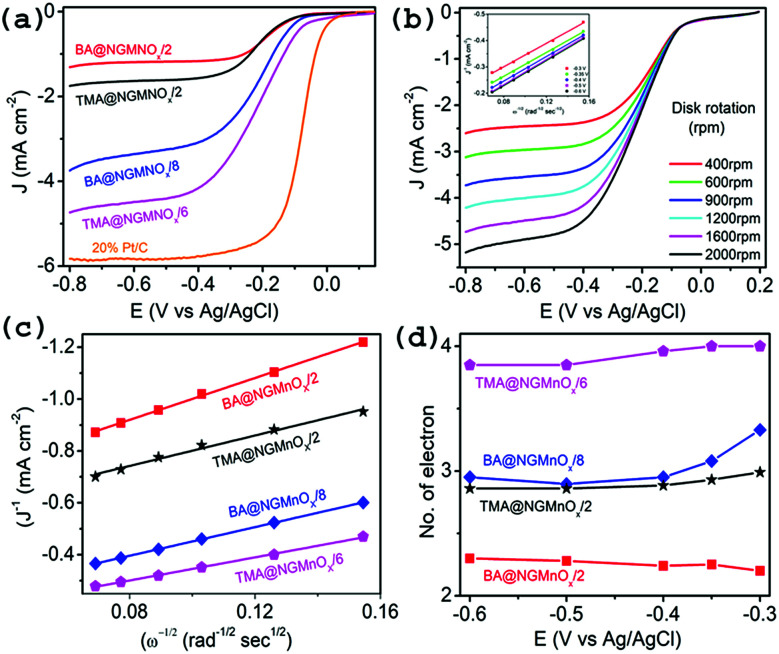
(a) LSV curves of TMA@NGMnO_*x*_/2, TMA@NGMnO_*x*_/6, BA@NGMnO_*x*_/2, BA@NGMnO_*x*_/8 and Pt/C catalysts on RDE in O_2_ saturated 0.1 M KOH solution with a rotation speed 1600 rpm. (b) LSV curves of TMA@NGMnO_*x*_/6 at different rotation speeds in O_2_ saturated 0.1 M KOH solution and the inset show K–L plots for TMA@NGMnO_*x*_/6 at different potentials. (c) K–L plots of TMA@NGMnO_*x*_/2, TMA@NGMnO_*x*_/6, BA@NGMnO_*x*_/2 and BA@NGMnO_*x*_/8 at −0.3 V potential and (d) number of transferred electrons ‘*n*’ for TMA@NGMnO_*x*_/2, TMA@NGMnO_*x*_/6, BA@NGMnO_*x*_/2 and BA@NGMnO_*x*_/8 catalysts at various potentials.

To obtain further insight into the ORR activities for hybrid catalysts, linear sweep voltammograms (LSVs) at different electrode rotation rates are taken in O_2_ saturated 0.1 M KOH electrolyte. Fig. 7a shows the LSV curves at 1600 rpm for TMA@NGMnO_*x*_/2, TMA@NGMnO_*x*_/6, BA@NGMnO_*x*_/2 and BA@NGMnO_*x*_/8 at scan rate of 10 mV s^−1^. The TMA@NGMnO_*x*_/6 shows highest electrocatalytic activity with onset potential −0.04 V *vs.* Ag/AgCl and ORR current density −4.17 mA cm^−2^ at −0.4 V followed by BA@NGMnO_*x*_/8, TMA@NGMnO_*x*_/2, and BA@NGMnO_*x*_/2 in the decreasing order of activity with onset potential and current density: −0.055 V, −3.09 mA cm^2^; −0.07 V, −1.58 mA cm^2^; and −0.072 V, −1.17 mA cm^−2^ respectively and is close to that of Pt/C. In our previous study on BA functionalized NG, the onset potential has been observed at −0.06 V which is ∼20 mV negative shift with respect to that of TMA@NGMnO_*x*_/6. Remarkably, the sample without MnO_*x*_ did not show current plateau at higher potential, which is a significant feature in all the MnO_*x*_ based samples. It may be noted that among the various NG@Mn_3_O_4_ hybrid samples reported in literature, TMA@NGMnO_*x*_/6 shows the most positive ORR onset potential and highest limiting current.^9,22,23^

The RDE polarographs for TMA@NGMnO_*x*_/6 (Fig. 7b) at electrode rotation rates of 400, 600, 900, 1200, 1600 and 2000 rpm show that the ORR current increases with increasing rotation rate. Fig. 7b (inset) show the Koutecky–Levich (K–L) plot for TMA@NGMnO_*x*_/6 at −0.3, −0.35, −0.4, −0.5 and −0.6 V, which is derived from the RDE data by applying K–L equation:

a 1/*J* = 1/*J*_L_ + 1/*J*_K_

*J*_L_ = *Bω*^1/2^*J*_K_ = *nFkC*_0_

b *B* = 0.62*nFC*_0_(*D*_0_)^2/3^*v*^−1/6^

where, *J* represents the disc current density, *J*_K_ and *J*_L_ are the kinetic and diffusion-limiting current densities respectively, *ω* is the angular velocity, *n* is the electron transfer number per O_2_ molecule, *F* is the Faraday constant (96 485 C mol^−1^), *C*_0_ is the bulk concentration of O_2_ (1.2 × 10^−6^ mol cm^−3^), *D*_0_ is the diffusion coefficient of O_2_ in the 0.1 M KOH electrolyte (1.9 × 10^−5^ cm^2^ s^−1^), is the kinetic viscosity of the electrolyte (0.01 cm^2^ s^−1^), *B* is the slope and *k* is the electron-transfer rate constant.^43^ The constant 0.62 is adopted when the rotation speed is expressed in radian per s. K–L plots (*J*^−1^*vs. ω*^−1/2^) are analyzed at various electrode potentials. The slops of their best fit lines are used to calculate the number of electron-transfer (*n*) on the basis of the K–L equation. The K–L plots for TMA@NGMnO_*x*_/6 at different potentials (inset Fig. 7b) show good linearity and near parallelism of the straight lines, which indicate a first order ORR kinetics. The RDE measurements at different rotation rates for TMA@NGMnO_*x*_/2, BA@NGMnO_*x*_/2 and BA@NGMnO_*x*_/8 and their corresponding K–L plots are given in Fig. S8.†

Depending on the efficiency of the catalyst, ORR proceeds through two alternate pathways: either a two-electron process or the most efficient four-electron process. In the two-electron pathway, O_2_ is reduced to H_2_O_2_, which further disproportionate to H_2_O and O_2_. On the other hand, in the most efficient four-electron pathway O_2_ is directly reduced to H_2_O. Fig. 7c and d show the linear fitted K–L plots at −0.3 V and the *n* values at different potentials respectively for TMA@NGMnO_*x*_/2, TMA@NGMnO_*x*_/6, BA@NGMnO_*x*_/2 and BA@NGMnO_*x*_/8. All the samples show minimal variation in *n* value at different potentials. The *n* value at −0.3 V for TMA@NGMnO_*x*_/6, TMA@NGMnO_*x*_/2, BA@NGMnO_*x*_/2 and BA@NGMnO_*x*_/8 are 4, 3, 2.2 & 3.3 respectively. These studies show that among the samples, TMA@NGMnO_*x*_/6 manifest best ORR activity in terms of onset potential, current density and electron transfer number. The highly efficient ORR activity for TMA@NGMnO_*x*_/6 may be correlated to its well dispersed MnO_*x*_ nanostructures on NG. Moreover, the highest amount of Mn^+3^ and Mn^+4^ in TMA@NGMnO_*x*_/6, among the studied samples may also have played decisive role for its ORR activity.

The durability of BA@NGMnO_*x*_/8, TMA@NGMnO_*x*_/6 and 20% Pt/C catalysts towards ORR are evaluated through *i*–*t* chronoamperometric method at −0.22 V in O_2_ saturated 0.1 M KOH solution with a rotation speed of 1600 rpm. Fig. S9† shows stability of the catalyst during the test for 12 000 s which indicates that the current density of the two studied materials decreases quickly at initial stage and becomes relatively steady after 1000 s. The catalyst BA@NGMnO_*x*_/8 and TMA@NGMnO_*x*_/6 show excellent stability by retaining 86 and 77% of its initial current density respectively which is better than that of commercial Pt/C.

All these results indicate that the duration of reaction in both TMA and BA functionalized growth affects the morphology of MnO_*x*_ crystals, surface Mn valency and subsequently the ORR activity. Previous studies showed that Mn(iv)–Mn(iii) couple in MnO_*x*_ surface acts as mediator for charge transfer to molecular O_2_, which in turn facilitate ORR activity.^31–33^ It may be noted that both in TMA and BA functionalized growth the ratio of Mn(iv) and Mn(iii) gradually increases with increase in reaction time (Table 1), which is responsible for the enhanced ORR activity in TMA@NGMnO_*x*_/6 and BA@NGMnO_*x*_/8. Moreover, among all the studied samples the most efficient ORR activity in TMA@NGMnO_*x*_/6 may be due to the highly uniform distribution of MnO_*x*_ nanostructures. However, the ORR activity decreases on further increasing the reaction time beyond 6 h (Fig. S10†), which is due to the observed dissolution of MnO_*x*_. However, CV and RDE studies show that the ORR activity of samples prepared in lower concentration of TMA follow the trend 0.5TMA@NGMnO_*x*_/12 > 0.5TMA@NGMnO_*x*_/8 > 0.5TMA@NGMnO_*x*_/4 (Fig. S11†). Similarly, for growth in presence of BA, highest ORR activity is observed in BA@NGMnO_*x*_/8 and decrease in activity is evident in BA@NGMnO_*x*_/12 (Fig. S12†). The study shows that in case of MnO_*x*_-NG heterostructure based electrocatalysts the functional molecule and reaction time plays pivotal role in getting the optimum ORR activity.

## Conclusions

In summary, we demonstrated evolution of various MnO_*x*_ phases and nanostructures on NG as the reactions proceed in presence of TMA or BA and studied their ORR activity. The functional molecule (TMA/BA), its concentration in the reaction mixture and duration of reaction are found to affect the phase and morphology of MnO_*x*_, which in turn affect their ORR activity. In case of TMA as functional molecule agglomerated particles of MnOOH were formed at 2 h of reaction but as reaction proceeded it transformed to well disperse 4–7 nm particles at 6 h. The highest ORR activity among all the studied samples was recorded for TMA@NGMnO_*x*_/6, in terms of positive onset potential, higher limiting current density and selective 4-electron pathway. At lower TMA concentration a completely different growth pattern was observed with continuous increase in ORR activity with reaction time up to 12 h. In presence of BA, MnOOH nano-flecks and nanorods are formed at 2 h of reaction, however after 8 h of reaction ∼5 nm diameter MnOOH nanowires and oval shaped Mn_3_O_4_ particles are formed. The ORR activity for BA@NGMnO_*x*_/8 was better than other samples in the series. On further correlating the XPS studies and ORR analysis, it was observed that the samples with higher amount of Mn(iv) and Mn(iii) on the surface showed better activity.

## Conflicts of interest

There are no conflicts to declare.

## Supplementary Material

RA-009-C9RA04677A-s001
