# Supplementary material for: Functional molecule guided evolution of MnOx nanostructure patterns on N-graphene and their oxygen reduction activity
Source: RSC Adv. 2019 Sep 4;9(48):27945–52. doi: 10.1039/c9ra04677a (PMC9071008; doi:10.1039/c9ra04677a)
Supplement: RA-009-C9RA04677A-s001 [file RA-009-C9RA04677A-s001.pdf]

Electronic Supplementary Material (ESI) for RSC Advances.
This journal is © The Royal Society of Chemistry 2019

Supporting Information

Functional Molecule Guided Evolution of MnO_x Nanostructure Patterns on N-Graphene and Their Oxygen Reduction Activity

Nibedita Behera, Swarna P. Mantry, Biswaranjan D. Mohapatra, Rajesh K. Behera and Kumar S. K. Varadwaj*

Department of chemistry, Ravenshaw University, Cuttack, Odisha-753003, India.

*E-mail: skvaradwaj@ravenshawuniversity.ac.in

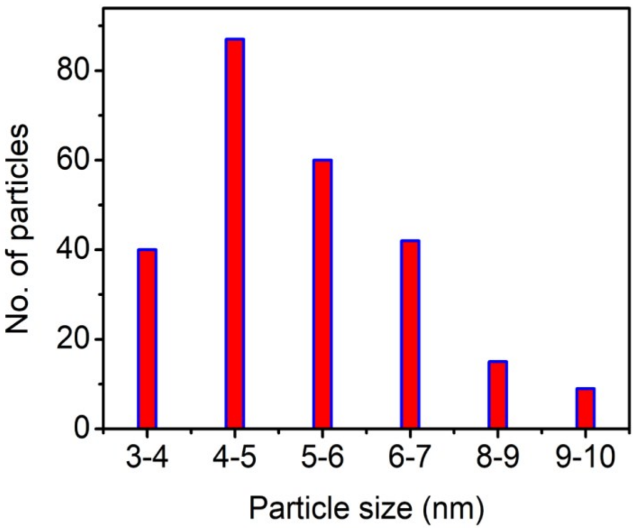

Figure S1. Particle size distribution histogram of TMA@NGMnO_x/6.

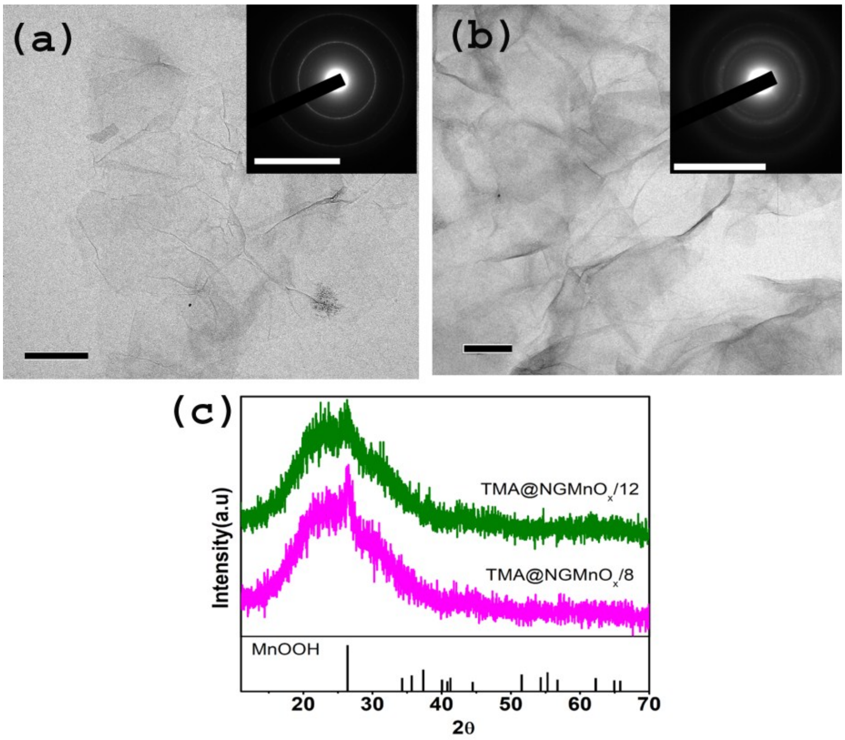

Figure S2. TEM images of (a) TMA@NGMnO_x/8 and (b)TMA@NGMnO_x/12 (scale bar: 200 nm) and the corresponding SAED patterns (insert) (scale bar: 10 1/nm).(c) The XRD patterns of TMA@NGMnO_x/8 and TMA@NGMnO_x/12.

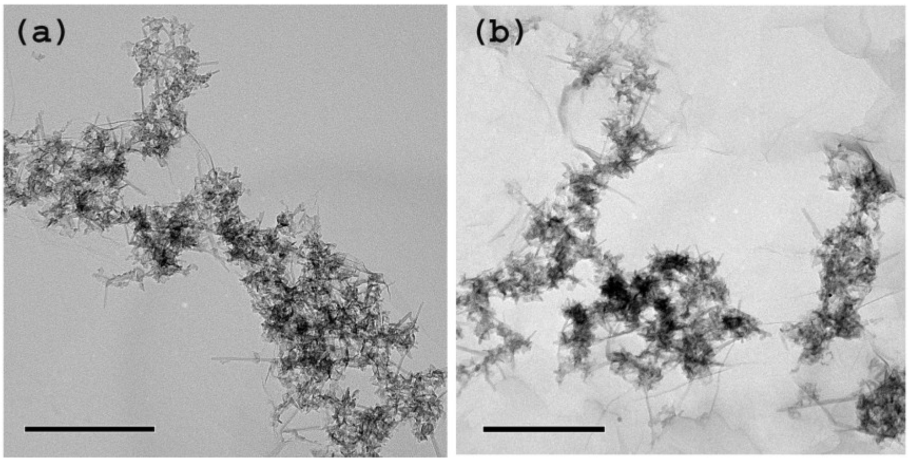

Figure S3. TEM images of (a) 0.5TMA@NGMnO$_x$/8 and (b) 0.5TMA@NGMnO$_x$/12 (scale bar: 200nm).

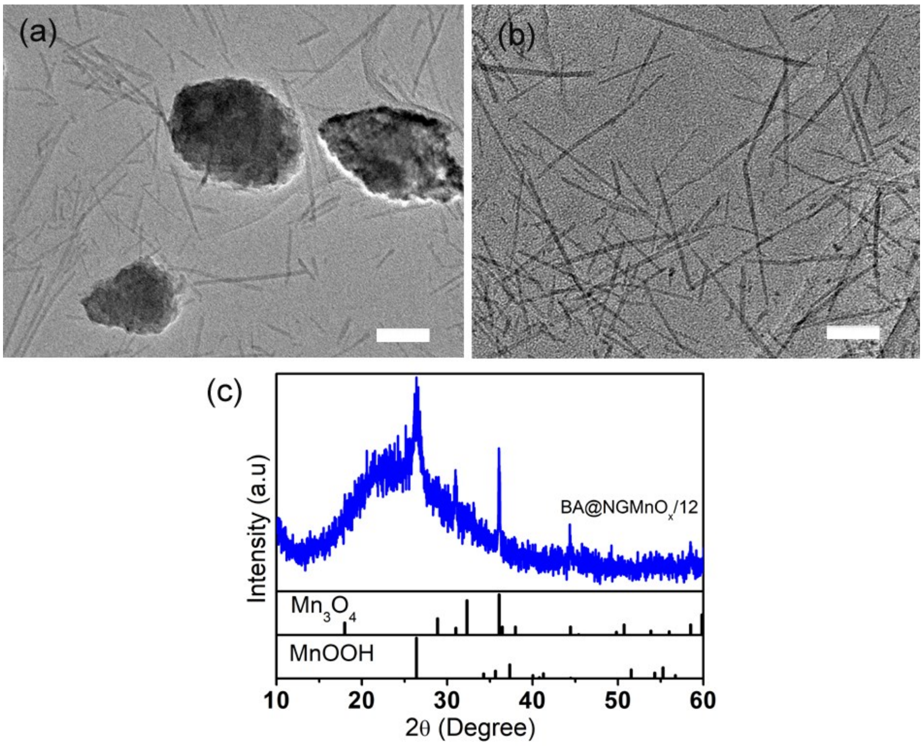

Figure S4. TEM images of BA@NGMnO$_x$/12 (a) Oval and (b) Wire structures (scale bar: 50 nm). (c) The XRD patterns of BA@NGMnO$_x$/12.

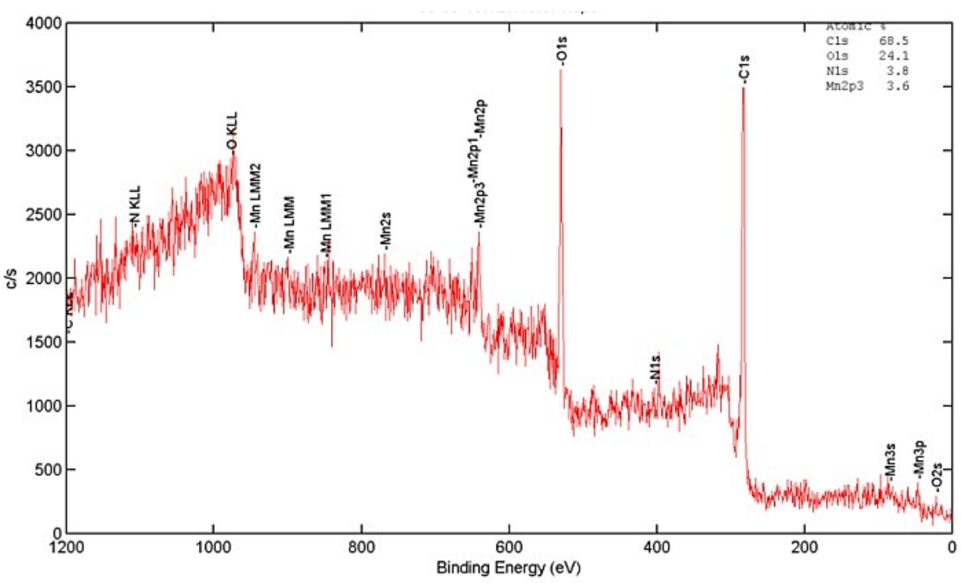

Figure S5. Survey scan of TMA@NGMnO$_x$/6 hybrid with atomic percentage.

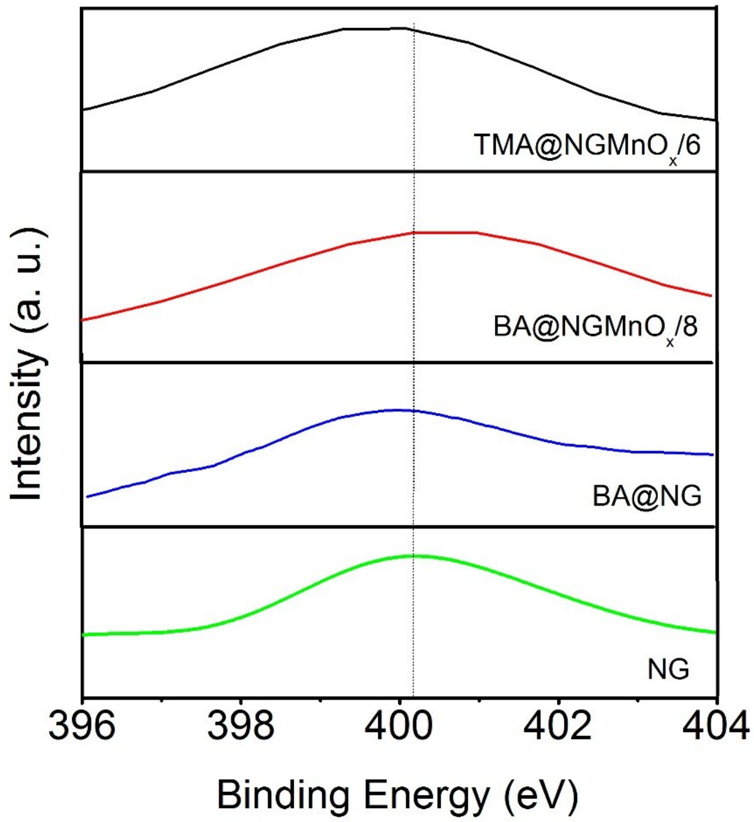

Figure S6. Survey scan N 1s XPS spectra for NG, BA@NG, BA@NGMnO$_X$/8 and TMA@NGMnO$_X$/6 hybrids.

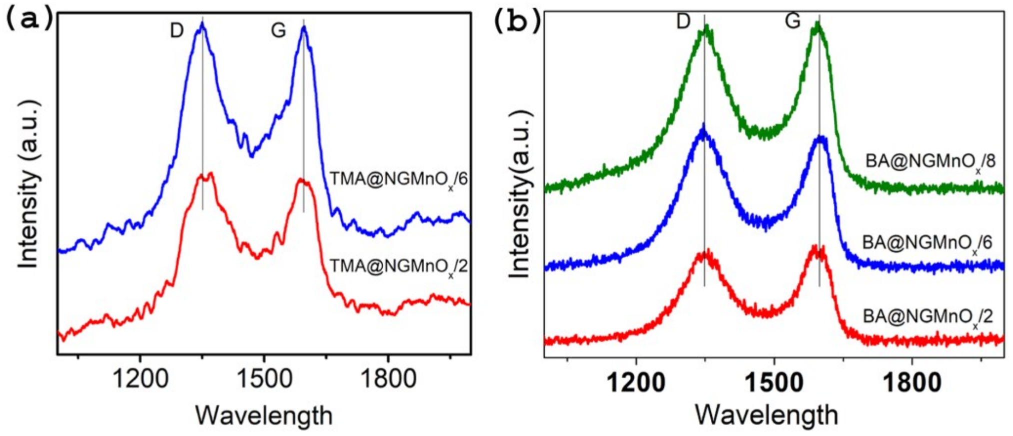

Figure S7. Raman spectra of (a) TMA@NGMnO$_x$/2 and TMA@NGMnO$_x$/6 (b) BA@NGMnO$_x$/2, BA@NGMnO$_x$/6 and BA@NGMnO$_x$/8 hybrids.

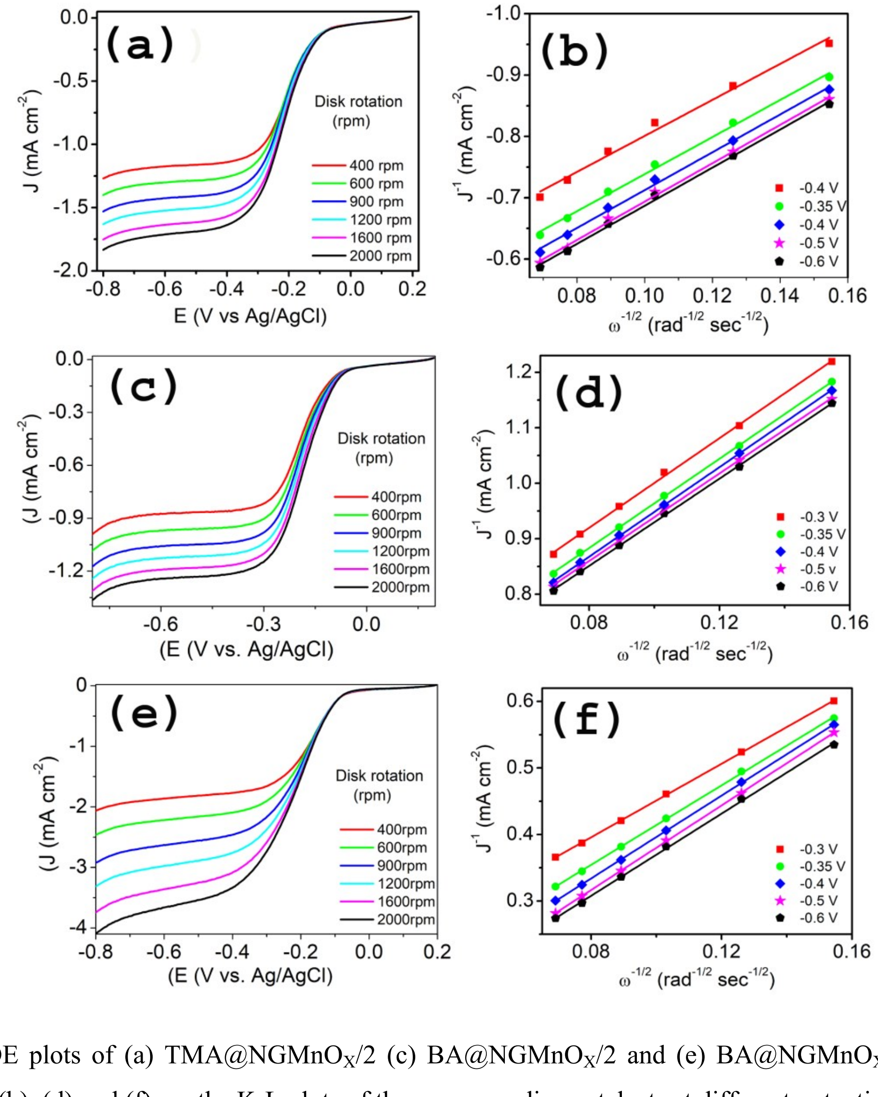

Figure S8. RDE plots of (a) TMA@NGMnO$_X$/2 (c) BA@NGMnO$_X$/2 and (e) BA@NGMnO$_X$/8 at different rotation speed. (b), (d) and (f) are the K-L plots of the corresponding catalysts at different potentials.

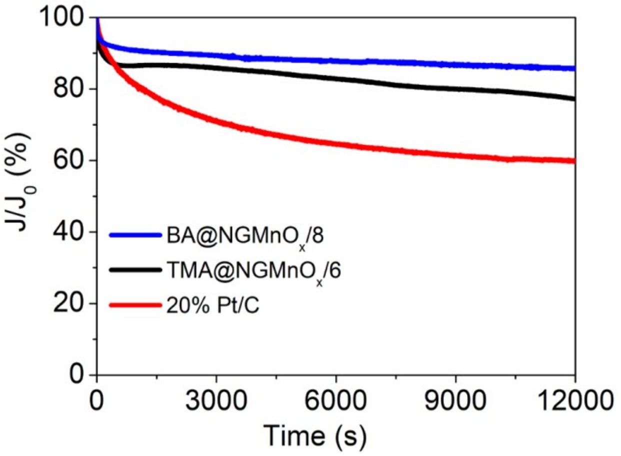

Figure S9. Chronoamperometric responses of BA@NGMnO$_x$/8, TMA@NGMnO$_x$/6 and 20% Pt/C catalysts at -0.22 V in O_2 saturated 0.1 M KOH, which are normalised to initial current responses.

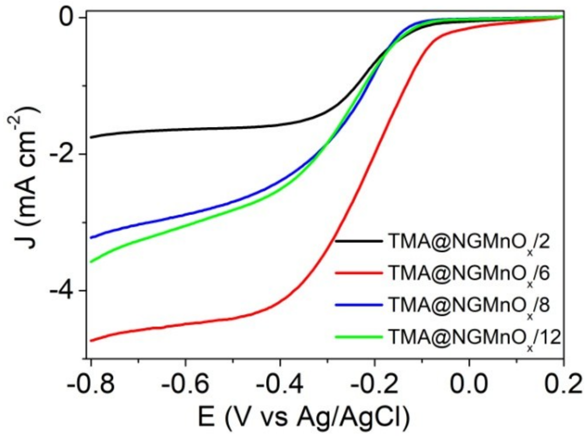

Figure S10. LSV curves of TMA@NGMnO$_x$/2, TMA@NGMnO$_x$/6, TMA@NGMnO$_x$/8 and TMA@NGMnO$_x$/12 catalysts on RDE in O_2 saturated 0.1 M KOH solution with a rotation speed of 1600 rpm at a scan rate of 10 mV s^{-1}.

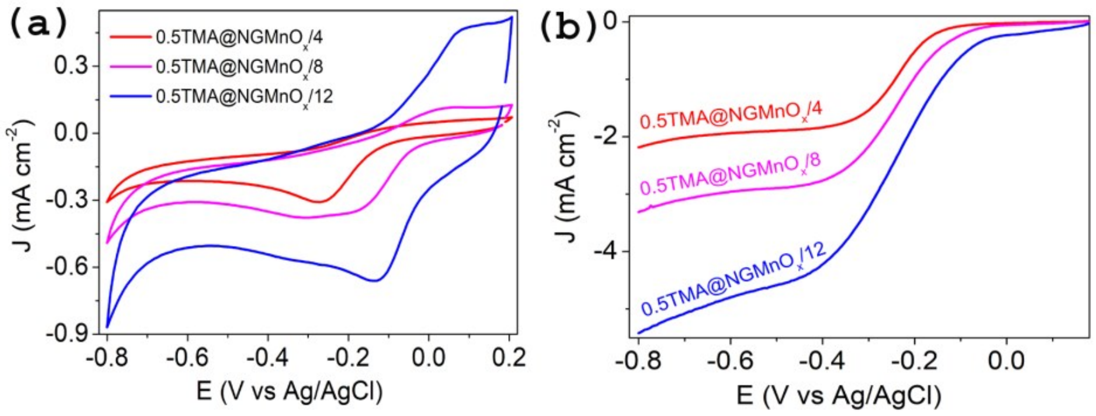

Figure S11. (a) Cyclic voltammetry study (b) LSV plots at a rotation speed of 1600 rpm for 0.5TMA@NGMnO$_x$/4, 0.5TMA@NGMnO$_x$/8 and 0.5TMA@NGMnO$_x$/12 nanohybrids, obtained from O_2 saturated 0.1 M KOH solution at a scan rate of 10 mV/sec.

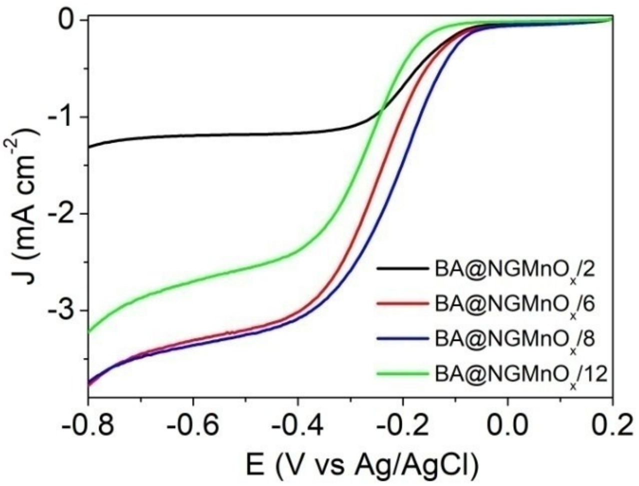

Figure S12. LSV curves of BA@NGMnO$_x$/2, BA@NGMnO$_x$/6, BA@NGMnO$_x$/8 and BA@NGMnO$_x$/12 catalysts on RDE in O_2 saturated 0.1 M KOH solution with a rotation speed of 1600 rpm at a scan rate of 10 mV/sec.
